# *Corynebacterium *endocarditis species-specific risk factors and outcomes

**DOI:** 10.1186/1471-2334-7-4

**Published:** 2007-02-06

**Authors:** Jaime Belmares, Stephanie Detterline, Janet B Pak, Jorge P Parada

**Affiliations:** 1 Section of Infectious Diseases, Louisiana State University Health Sciences Center. 2390 West Congress Street, Lafayette, LA. USA; 2 Division of General Internal Medicine. Loyola University Chicago Medical Center. 2160 South First Avenue. Maywood, IL. USA; 3 Medicine-Neurology Service Line, Hines VA Hospital. Hines, IL. USA; 4 Midwest Center for Health Services and Policy Research. Hines, IL. USA

## Abstract

**Background:**

*Corynebacterium *species are recognized as uncommon agents of endocarditis, but little is known regarding species-specific risk factors and outcomes in *Corynebacterium *endocarditis.

**Methods:**

Case report and Medline search of English language journals for cases of *Corynebacterium *endocarditis. Inclusion criteria required that cases be identified as endocarditis, having persistent *Corynebacterium *bacteremia, murmurs described by the authors as identifying the affected valve, or vegetations found by echocardiography or in surgical or autopsy specimens. Cases also required patient-specific information on risk factors and outcomes (age, gender, prior prosthetic valve, other prior nosocomial risk factors (infected valve, involvement of native versus prosthetic valve, need for valve replacement, and death) to be included in the analysis. Publications of *Corynebacterium *endocarditis which reported aggregate data were excluded. Univariate analysis was conducted with chi-square and t-tests, as appropriate, with p = 0.05 considered significant.

**Results:**

129 cases of *Corynebacterium *endocarditis involving nine species met inclusion criteria. *Corynebacterium *endocarditis typically infects the left heart of adult males and nearly one third of patients have underlying valvular disease. One quarter of patients required valve replacement and one half of patients died. Toxigenic *C. diphtheriae *is associated with pediatric infections (p < 0.001). Only *C. amycolatum *has a predilection for women (p = 0.024), while *C. pseudodiphtheriticum *infections are most frequent in men (p = 0.023). *C. striatum*, *C. jeikeium *and *C. hemolyticum *are associated with nosocomial risk factors (p < 0.001, 0.028, and 0.024, respectively). No species was found to have a predilection for any particular heart valve. *C. pseudodiphtheriticum *is associated with a previous prosthetic valve replacement (p = 0.004). *C. jeikeium *infections are more likely to require valve replacement (p = 0.026). Infections involving toxigenic *C. diphtheriae *and *C. pseudodiphtheriticum *are associated with decreased survival (p = 0.001 and 0.032, respectively).

**Conclusion:**

We report the first analysis of species-specific risk factors and outcomes in *Corynebacterium *endocarditis. In addition to species-specific associations with age, gender, prior valvular diseases, and other nosocomial risk factors, we found differences in rates of need for valve replacement and death. This review highlights the seriousness of these infections, as up to 28% of patients required valve replacement and 43.5% died.

## Background

*Corynebacterium *species, or "diphtheroids", are aerobic, non-sporulating, pleomorphic, Gram-positive bacilli that are often considered non-pathogenic components of normal skin flora and mucosal membranes. Although frequently isolated in cultures, they are commonly and often justifiably assumed to be contaminants [1,2]. Nevertheless, the ability of these bacteria to cause life-threatening disease is well established, and over the last decade there have been increasing reports of their pathogenic potential in numerous clinical scenarios, including bacteremia and endocarditis [3-5]. Diphtheroids are now thought to cause 9% of early and 4% of late prosthetic valve endocarditis (PVE), [1,4] but only 0.2 to 0.4% of cases of native valve endocarditis (NVE) [4,6]. Recognized risk factors for *Corynebacterium *endocarditis include preexisting cardiac disease, a prior history of bacterial endocarditis, and the presence of prosthetic devices, including intravascular access and auriculo-ventricular cerebrospinal fluid shunts [1,6,7]. It is unknown whether certain species of *Corynebacterium *are more frequently associated with endocarditis, are associated with different predisposing conditions or risk factors, or if they have differences in outcomes – such as requiring valve replacement surgery or greater mortality. To date, most published reports of *Corynebacterium *endocarditis have been case reports and series, and no detailed species-specific analysis of risk factors and outcomes in *Corynebacterium *endocarditis has been conducted. Herein, we review the literature for patient-level reports of *Corynebacterium *endocarditis and conducted the first species-specific analysis of risk factors and outcomes in *Corynebacterium *endocarditis.

## Methods

A Medline search of English language journals for reported cases of *Corynebacterium *endocarditis. In those reports with case series, every effort was made to obtain the primary reference or discuss the particulars of the case with the original authors. We standardized our definitions for inclusion/exclusion to include only cases with persistent *Corynebacterium *bacteremia (at least >1 positive blood culture with an identified *Corynebacterium *species), murmurs described by the authors of the source publication as identifying the affected valve, or vegetations found either in an echocardiographic report, or in surgical or autopsy specimens. A number of cases described *Corynebacterium *bacteremia in the setting of pacemaker infections and non-valvular endovascular foci of infections. While these are considered by many to be "endocarditis equivalents", they did not meet our definition of endocarditis and were dropped from the analysis. In addition, only cases whose source report included individual patient-specific information on risk factors and outcomes were included in the analysis. Patient-level characteristics and medical information of interest are detailed below. However, publications of *Corynebacterium *endocarditis which reported aggregate data were excluded.

Patient characteristics in the analysis included demographic data, such as gender and age. Pediatric cases were defined as those occurring in patients less than 18 years old of age and adult cases as those occurring in patients 18 years or older. Medical information recorded included the presence of comorbid illnesses, pre-existing valvular disease other than valve replacement, infected valve, and involvement of native versus prosthetic valve. Nosocomial risk factors were defined as the presence of prosthetic medical devices other than artificial heart valves, such as pacemakers, ventriculoperitoneal shunts, and the use of intravascular devices. Primary outcomes of interest were need for valve replacement and mortality. Nomenclature for *Corynebacterium *species was based on those approved by the International Code of Nomenclature of Bacteria and species identification was classified as reported in the original source document [8]. Statistical analysis was done using SPSS^® ^version 13.0 (Chicago, IL). Univariate analysis was conducted with chi-square and t-tests, as appropriate, with p = 0.05 (two sided) was considered statistically significant. In three cases, *Corynebacterium *endocarditis was found to have only a single case for that unique species. These were excluded from the analysis because of their small numbers simultaneously added little to the analysis and because of the potential bias from the intrinsic marked skew that results from inclusion of data where a single case provides 100% of the data points for an entire species. Study received approval of the Institutional Review Board and was conducted in accordance with the Helsinki declaration.

### Case Report

A 62 year-old man with a history of hypertension, alcoholism, and mild renal insufficiency, presented to our hospital with a two-day history of fevers, lower back pain, and new onset of urinary retention. One week prior to admission, the patient had undergone diagnostic cardiac catheterization for new onset of atrial fibrillation and had been started on coumadin. Other medications prior to admission were digoxin, diltiazem, furosemide, lisinopril, metoprolol, omeprazole, simvastatin, coumadin. Vital signs on admission were: blood pressure 100/60, temperature 101.4^°^F, heart rate 100, respiratory rate 20, He had an irregular heart rhythm without murmurs and mild right flank pain. There was a small non-tender and non-purulent ecchymotic area in the right groin at the site of previous catheterization. His neurologic exam was nonfocal except for new 4/5 bilateral lower extremity weakness. White blood cell count was 18.4 K/mm^3^, with 75% neutrophils and 12% bands. Hemoglobin and platelets were normal. Sodium 128 mmol/L, blood urea nitrogen 46 mmol/L, creatinine 1.7 mmol/L. INR was 11.21. Amylase and lipase were 557 and 325. A chest x-ray, and a computed tomography (CT) scan of the lumbar spine were unremarkable. A CT scan of the abdomen showed mild early pancreatitis without dilatation of the biliary tree. Blood and urine cultures were sent and the patient was diagnosed with mild pancreatitis and coagulopathy secondary to coumadin overdose. He was was started on intravenous levofloxacin 500 mg, with resolution of the fever by the next day. He received vitamin K and his coumadin was temporarily stopped.. Over the next days, his INR decreased to 1.3 and his abdominal pain and leukocytosis improved, but the back pain and urinary retention continued. By the sixth day of admission, he complained of stool incontinence. A magnetic resonance image (MRI) study of the lumbar spine showed inflammatory diskitis with fluid in the L5/S1 disc space, along with osteomyelitis and a posterior epidural abscess with moderate-to-severe vertebral canal stenosis. The Spine Surgery Service was consulted, but they felt that the neurological findings were not explained by the lesion seen on MRI and recommended non-operative management.

Blood cultures from admission later grew Gram-positive bacilli, and repeat cultures confirmed *Corynebacterium striatum *bacteremia on the sixth day of admission. At this time, antibiotics were changed to vancomycin 1500 mg iv daily with subsequent improvement of the back pain, urinary retention and stool incontinence. Follow up blood cultures were negative. A transesophageal echocardiogram (TEE) revealed moderate aortic insufficiency and a 3–4 mm vegetation on the non-coronary cusp of the aortic valve, with a perforation at the same site. Cardiovascular surgery recommended that a valve replacement be deferred pending initial antibiotic treatment. A follow-up MRI of the spine reported no significant change on the fluid collection. Surgery was offered, but the patient refused stating he felt decreased weakness and wanted to pursue a conservative management. Over the next days, the patient showed progressive improvement in his lower extremity strength and was able to ambulate with the help of a physical therapist. His urinary retention and bowel dysfunction improved as well.

Three weeks later, the patient became febrile and acutely dyspneic. An emergent TEE showed an increase in the regurgitant jet through the aortic valve perforation. The patient underwent emergency coronary aortic bypass grafting and aortic valve replacement. Pathology of the aortic valve revealed a heavily calcified aortic valve, and the gram stain showed gram positive coccobacilli. Cultures of the valves were negative, however, the valve had been mistakenly placed on formalin prior to submission to the Microbiology Laboratory. The patient did well postoperatively and was discharged on intravenous vancomycin. At a two-month follow-up appointment the patient's back pain had resolved and an MRI showed resolution of the epidural abscess. Post treatment surveillance blood cultures were sterile after a total of 12 weeks of intravenous vancomycin after his discharge from the hospital. At follow up over two years later the patient remained infection free.

## Results

Our search returned 172 cases of *Corynebacterium *endocarditis with meaningful patient-level clinical data. Of these, 43 (25.0%) were excluded (Table 1). In 21 cases (48.8%) the isolates were not identified to a species level, 14 cases (32.5%) they were identified only as "diphtheroids", and seven cases (16.3%) were single or double cases identified only by their CDC group (Table 1). *C. tuscaniae*, *C. accolens*, *and C. pyogenes *were excluded as they each contributed only a single case and were not amenable to analysis. Fourteen cases (32.5%) were excluded because they lacked documentation identifying which specific valve was infected or information on our primary outcomes (valve replacement and death). Five cases (11.6%) were infections of pacemakers, prosthetic devices other than heart valves, or involved non-heart valve locations.

**Table 1:** Excluded cases of *Corynebacterium *endocarditis (N = 43)

**Non speciated *Corynebacterium *and isolates not yet recognized as a species**	
"Diphtheroids"	14 (32.5)
*Corynebacterium *CDC group 1	2 (4.6)
*Corynebacterium *CDC group A4	1 (2.3)
*Corynebacterium *CDC group I1	1 (2.3)
*Corynebacterium *CDC group D2	1 (2.3)
*Corynebacterium *CDC group G1	1 (2.3)
*Corynebacterium *CDC group G2	1 (2.3)
**Only one case identified by species**	
*C. tuscaniae*	1 (2.3)
*C. accolens*	1 (2.3)
*C. pyogenes*	1 (2.3)
Missing primary study outcomes*	13 (30.2)
**Special cases†**	5 (11.6)
**No valve identified**	1 (2.3)

**TOTAL**	**43 (100)**

* Valve replacement, death.

† Special cases: pacemaker infections (two cases), vegetation on the posterior wall of the right atrium (one case), vegetation on the proximal part of the right pulmonary artery but not on the pulmonic valve (one case), infection of an aortic allograft placed during a repair of a tetrallogy of Fallot (one case).

129 cases (75.0%) had sufficiently detailed patient-level information to be included in the final analysis. Details of the clinical and epidemiological findings of these cases are found in Table 2 [[6-96],97]. Species encountered included 60 cases (46.5%) of non-toxigenic *C. diphtheriae *(NTCD), 18 cases (14.0%) of *C. pseudodiphtheriticum*, 14 cases (10.9%) of *C. striatum*, 14 cases (10.9%) of toxigenic *C. diphtheriae *(TCD), 13 cases (10.1.%) of *C. jeikeium*, and four cases (3.1%) of *C. xerosis*. Finally, *C. amycolatum*, *C. minutissimum*, and *C. hemolyticum *each were responsible for two cases of endocarditis.

**Table 2:** *Corynebacterium *endocarditis: risk factors and outcomes by *Corynebacterium *species.

Species & references	Number of cases† (% total)	Mean age (years)	Male n (%)	Left sided Endocarditis^‡^ n (%)	Previous prosthetic valve n (%)	Previous valvular disease^§^ n (%)	Nosocomial risk factors^||^ n (%)	Required valve replacement n (%)	Survived n(%)
*Non-toxigenic C. diphtheriae *5, 20, 22, 24–26, 27, 32, 46, 48–52, 54, 62, 63, 65, 66, 68, 78, 94	60 (46.5%)	56 ± 13.8 **p = 0.012**	45 (75%)	58 (95.1%)	8 (13.3%)	17 (28.3%)	1 (1.7%) **p = 0.017**	17 (28.3%)	39 (65%) p = 0.060
*C. pseudo-diphtheriticum *18, 20, 36–38, 40, 42–45, 81, 83	18 (14.0%)	45 ± 21.32	17 (93.8%) **p = 0.023**	18 (100%)	8 (44.4%) **p = 0.004**	6 (33.3%)	0 (0%)	6 (37.5%)	6 (33.3%) **p = 0.032**
*Toxigenic C. diphtheriae *23, 27, 29, 49, 64, 66	14 (10.9%)	17.5 ± 16.5 **p < 0.001**	8 (57.1%)	12 (85.7%)	1 (7.1%)	2 (14.3%)	0 (0%)	0 (0%) *p = 0.014	2 (14.3%) **p = 0.001**
*C. striatum *6, 7, 9–15, 53, 56, 62	14 (10.9%)	61.3 ± 13.9 **p = 0.018**	7 (50%)	12 (85.7%)	3 (21.4%)	4 (28.6%)	5 (35.7%) **p < 0.001**	4 (28.6%)	11 (78.6%) p = 0.074
*C. jeikeium *6, 16, 19, 25, 34, 35, 69, 79, 93	13 (10.1%)	57.2 ± 13.6 **p = 0.024**	10 (76.9%)	12 (92.3%)	5 (38.5%) p = 0.064	6 (42.6%)	3 (23.1%) **p = 0.028**	7 (53.8%) **p = 0.026**	10 (76.9%)
*C. xerosis *59, 75, 82, 95	4 (3.1%)	51.2 ± 12.3	3 (75%)	4 (100%)	0 (0%)	2 (50%)	0 (0%)	2 (50%)	2 (50%)
*C. hemolyticum *77, 80	2 (1.6%)	68.5 ± 26.1	2 (100%)	2 (100%)	0 (0%)	1 (50%)	1 (50%) **p = 0.024**	0 (0%)	0 (0%)
*C. minutissimum *86, 91	2 (1.6%)	35.5 ± 6.3	1 (50%)	2 (100%)	0 (0%)	1 (50%)	0 (0%)	0 (0%)	2 (100%)
*C amycolatum *6, 60	2 (1.6%)	81 ± 9.8	0 (0%) **p = 0.024**	2 (100%)	0 (0%)	1 (50%)	0 (0%)	0 (0%)	1 (50%)

Total	129 (100%)	35.7 ± 22.9	93 (72.1%)	123 (94.6%)	25 (19.3%)	40 (31.0%)	10 (7.7%)	36 (27.9%)	73 (56.6%)

*Data derived from References 2–97 (specific citations listed in by-species citations, column 1). All *p *values are compared to the mean.

† Percentages of the total number of cases (within column 2). Note: All other percentages represent values for each *Corynebacterium *species (percentage within each row).

‡ Left sided endocarditis: Aortic or mitral valve endocarditis.

§Previous valvular disease other than valve replacement.

|| Nosocomial risk factors: presence of an intravascular access device, dialysis fistula, pacemaker, or presence of a prosthetic device other than a valve

Patients' age ranged from 4 to 88 years (mean age 35.7 ± 22.9), and 93 (72.1%) were male.

Among all cases, previous valvular disease was present in 40 (31.0%) of the cases, including a previous valvular replacement in 25 cases (19.3%). Predisposing nosocomial risk factors were present in ten patients (7.7%). Left-sided endocarditis was present in 94.6% of the cases. Therapeutic valve replacement was required in 36 cases (27.9%). Mortality attributable to *Corynebacterium *endocarditis infection was high, with 56 patients (43.4%) succumbing to their infection.

*C. amycolatum *infections were more frequent in women (100%, p = 0.024), while *C. pseudodiphteriticum *infections were more frequently found in men (93.8%, p = 0.023). Species-specific age-related associations were also noted. In general, most *Corynebacterium *species were more likely to cause endocarditis in adults (attaining statistical significance for *C. striatum*, *C. jeikeium*, and NTCD, p = 0.018, p = 0.024, and p = 0.012, respectively), while TCD was the only species that infected children more frequently (p < 0.001).

All of the species showed a predilection for left-sided endocarditis (Table 2), but no species had a statistically significant predilection for any particular valve. There were no significant differences among species' associations with a history of previous valvular disease other than in cases of prior valve replacement, where *C. pseudodiphtheriticum *was found to more commonly infect prosthetic valves (p = 0.004). *C. striatum*, *C. jeikeium*, and *C. hemolyticum *were more commonly associated with a nosocomial risk factors (p < 0.001, p = 0.028, p = 0.024, respectively). *C. jeikeium *endocarditis was also the most likely to require valve replacement as a result of the infection (53.8%, p = 0.026). Overall, valve replacement was required in 27.9% of cases, but there were no significant differences in the likelihood of need for valve replacement among the other species.

Overall survival in *Corynebacterium *endocarditis across all species was 56.6%. *C. striatum *was associated with the highest survival rate (78.6%, p = 0.074). In contrast, TCD and *C. pseudodiphtheriticum *were associated with a poorer survival than other species (14.3%, p = 0.001 and 33.3%, p = 0.032, respectively).

## Discussion

We conducted the largest detailed patient-level analysis of *Corynebacterium *endocarditis to date, and report the first species-specific associations for these infections. Foremost, we believe that our findings highlight the seriousness of *Corynebacterium *endocarditis, as over one quarter of patients required valve replacement and mortality was 43.4%. Overall,*Corynebacterium *endocarditis typically infects adult males and has a strong predilection for left sided involvement; nearly one-third of patients have underlying valvular disease.

Our study found significant differences in species-specific risk factors in *Corynebacterium *endocarditis, including age and gender associations, underlying clinical risk factors, predilection for prosthetic valves, as well as notable differences in clinical outcomes, such as need for valve replacement and survival.

The species-specific analysis revealed a strong gender predilection for two *Corynebacterium *species. *C. amycolatum *infections occurred exclusively in females while *C. pseudodiphteriticum *occurred overwhelmingly in males. Age-related species-specific findings were also noted. NTCD, *C. striatum *and *C. jeikeium *have significant predilection for adults. In contrast to NTCD, TCD is significantly more common in children, as consistent with previous literature and historic associations. In addition, adults are more likely than children to have a *Corynebacterium *endocarditis of nosocomial origin (p = 0.050), which is likely to reflect a greater likelihood of prosthetic medical devices, invasive procedures, and intravascular access in the adult population. While not reaching statistical significance, adults were also more likely to have endocarditis of a prosthetic valve (p = 0.073). This is likely to reflect a greater prevalence of prosthetic valves in the adult population.

Species-specific associations with a nosocomial origin were found for *C. striatum*, *C. jeikeium*, and *C. hemolyticum*. While these species are associated with nosocomial risk factors, they were not associated with a higher incidence of need for valve replacement or greater mortality. Of all *Corynebacterium *species, only *C. pseudodiphtheriticum *showed a predilection for prosthetic versus native valves. This species was also found to have a statistically significant increase in mortality, with two thirds of cases not surviving their infection. An unexpected finding is the greater probability of valve replacement (p = 0.014) and mortality (p = 0.001) found in TCD endocarditis. It is possible that this might be due, in part, to including older reports from time periods when antibiotic drugs and other therapeutic measures were unavailable or less effective fro managing endocarditis. However, even recent reports of TCD endocarditis have shown a high risk of complications and death even in the setting of previous immunization and modern antibiotic treatment regimens [52,64]. Of all *Corynebacterium *species, *C. striatum *endocarditis was associated with the greatest likelihood of survival, with more than three quarter of cases surviving the infection. In contrast, only one third of *C. pseudodiphtheriticum *cases, and one in six TCD cases survived.

There are several limitations to this study which should be considered. This search was limited to English language journals and did not include cases published in other languages. We recognize that classification of isolates and nomenclature for *Corynebacterium *may have changed over the time period that is encompassed by the source publications included in our analysis, and that new molecular analysis techniques have led to the reclassification of some isolates, and may impact our findings. Similarly, we recognize our acceptance of cases as endocarditis is based on source publication reports and we did not personally review the original microbiology, pathology, or imaging studies on these cases. These are well-recognized intrinsic limitations to retrospective studies and reviews of published data. While every effort was made for accuracy, we are limited to the information available in these published reports, and we can not predict how additional unpublished cases would affect our findings. This is equally true for the cases where insufficient detailed data forced exclusion from the analysis. It is possible that the associations described in this study could change as more information is available. This is especially true for those species that were least encountered but kept in our analysis (e.g., *C. hemolyticum*, *C. minutissimum*, *and C. xerosis*) or excluded because we could only identify a single case for this unique species (*C. tuscaniae*, *C. accolens*, and *C. pyogenes*).

## Conclusion

Our study reveals a number of hitherto fore unknown species-specific associations between risk factors and outcomes in *Corynebacterium *endocarditis, including for demographic factors (gender and age), clinical factors (nosocomial risk factors, prosthetic valves), as well as outcomes (need for valve replacement and survival). Clinicians would be wise to keep these organisms in mind as rare causes of endocarditis, particularly when managing patients who present with bacteremia due to gram positive rods.

## Competing interests

None of the authors have potential conflicts of interest to report, although JPP serves on the Speakers' Bureau of Pfizer and Elan Pharmaeuticals, and serves as site principal investigator for clinical trials sponsored by GlaxoSmithKline and Pfizer Pharmaceuticals

## Authors' contributions

JPP conceived and oversaw the study and manuscript development. JB and JBP contributed a new case report of *C. striatum *endocarditis. All authors contributed to the reviews and evaluated the case series report. JB and JPP developed the database, analyzed and interpreted the data. All authors contributed to the writing of the manuscript.

## Pre-publication history

The pre-publication history for this paper can be accessed here:


